# Individual goal-setting in municipal homecare: A participatory appreciative action and reflection study

**DOI:** 10.1177/22799036231181198

**Published:** 2023-06-13

**Authors:** Sofia Tavemark, Annica Kihlgren, Margaretha Norell Pejner, Inger James

**Affiliations:** 1 School of Health Sciences, Örebro University, Örebro, Sweden; 2 Örebro Municipality Healthcare and Social Services, Örebro, Sweden; 3 Research Environment: Older People’s Health and Living Condition, Örebro University, Örebro, Sweden; 4 Department of Home Care, Halmstad Municipality, Halmstad, Sweden; 5 Faculty of Health, Science and Technology, Karlstad University, Karlstad, Sweden

**Keywords:** Homecare, individual goal, municipality, action research, public health

## Abstract

**Background:**

There is a need for structural change in municipal homecare to shift power to older persons and to center the individuals in need. To make this change, the individual older persons should have enough self-determination to formulate their own individual homecare goals. Our aim was to explore how stakeholders reason about individual goal-setting in homecare.

**Design and methods:**

We theoretically and methodologically used a participatory appreciative action and reflection (PAAR) design. The stakeholders, that is, the older persons, the older persons’ relatives, and the multi-professional team, were seen as co-researchers. Data were collected between 2019 and 2020 through in depth-interviews, focus group discussions, and reference groups. The data were analyzed using thematic analysis.

**Results:**

We learned from the stakeholders that it was a struggle to sustain the individual’s goal to continue life as usual, that is, being an ordinary human being with an ordinary everyday life and maintaining individual roles. The individual wants to improve health, be active, and enjoying life. The individuals were struggling against the homecare organization, which tended to overshadow the individual’s goals. The individual’s goals fall under several legal jurisdictions and come to be overshadowed by the professionals’ dominant goal. The organization is rigid, with finances and resources creating the framework.

**Conclusion:**

We learned that older persons receiving homecare must have the same rights as other citizens in society, which is in line with public health goals.

## Introduction

Worldwide, we are living longer and getting older. This implies that older people with their accumulated knowledge and experience should increasingly be seen as assets to others and society. However, older people can have an increased risk of ill health, and multiple diseases can occur simultaneously. This, along with other life changes such as retirement, moving into more practical housing, as well as the deaths of partners, relatives, and friends, can have consequences in terms of declining health. It is important for society to meet this challenge and safeguard the health of older people.^1^ Health is defined as “a state of complete physical, mental and social well-being and not merely the absence of disease or infirmity. Health is a resource for everyday life, not the object of living. It is a positive concept emphasizing social and personal resources as well as physical capabilities.”^2^ Home healthcare (HHC) is important, as it is needed in order to maintain health during aging, so the overall goals of HHC must be stipulated and described.^3^

Aging in place, in which the individual can receive HHC to be able to stay at home, is recommended worldwide,^4–6^ and is in line with public health goals.^1,7^ It is not self-explanatory what HHC means,^8^ and the definition differs within and between countries. Internationally, there are different models for offering HHC (e.g. chronic care, integrated care, or disease-specific integrated care),^9^ which can be either tax-financed or carried out by private actors. In Sweden, municipal care for older people is split between HHC and homecare (HC). Swedish HC comprises a wide range of help with daily activities, including household chores, grocery shopping, personal hygiene, social support, and administration (e.g. documentation and making telephone calls for the individual). HHC comprises medical care, nursing, and rehabilitation, with registered nurses (RNs) and occupational therapists (OTs) being responsible and delegating medical and rehabilitation interventions to nurse assistants (NAs).^5^ Hereafter, HC is used to refer to both homecare and home healthcare. The older persons in Sweden can apply for assistance from the municipality to get help from HC in their daily life to be able to stay at home.^10^ During 2020, approximately 258,000 people received municipal HC in Sweden, of whom 236,000 were over 65 years of age.^11^ It is stipulated that care for older people be mainly financed through taxes,^10^ although the recipient is still charged various fees for HC services.^12^

Municipal HC professionals can take care of individuals and their families for up to several years on an individual basis^13^; some individuals need extensive and protracted care, while others have only short-term needs.^14^ It is important to know when older individuals need HC, so it can be provided in a timely manner.^15^ The process of getting HC starts with the case manager at the social service office, who assesses the individual’s needs based on, for example, the degree of dependency, and makes decisions about HC interventions according to these needs. The first-line managers then allocate the interventions to the administrative staff, who make a schedule, and then the NAs start offering support through home visits.

HC professionals are seen as the key team members, together with the older person and her/his relatives, whereas physicians and physiotherapists may be engaged on a consultation basis.^16^ The NAs are the professionals who spend the most time with the individual older persons and know them best; the NAs serve as eyes and ears, advocating for care needs to other professionals both in the team and outside municipal homecare.^17^ NAs also help maintain the older person’s self-determination and confirm the person’s self-image.^18^ Although national guidelines and local core values have been stipulated to ensure the dignity, well-being, and meaningful everyday life of older persons,^19^ older persons still find it difficult to influence their own care.^20^ There is a need for structural change in municipal elderly care^16^ to shift power to the older person and center the individuals in need.^11^ To make this change, the individual older persons should have sufficient self-determination to formulate their goals within the HC provided. Many stakeholders are involved in the individual’s HC; in this study, these include the individual himself/herself, relatives, and multi-professional teams within HC. The aim was accordingly to explore how these stakeholders reason about individual goal-setting in daily life within HC.

## Design and method

This study was part of a larger project examining a municipality in central Sweden that implemented a structural change program to address individual goals in HC. To learn from those who have experience of HC and to allow their voices to be heard, participant-based research with a bottom–up perspective was conducted.^21^ We theoretically and methodologically used a participatory appreciative action and reflection (PAAR) design to find out about individual goalsetting. This method allows the stakeholders to be seen as equal actors, co-researchers, in the process. The PAAR design is an iterative method with cycles of action and reflection, focusing on opportunities, creative solutions, and suggestions to reduce the obstacles.^22^

### Stakeholders

Convenient sampling was completed, resulting in six HC units and 81 stakeholders included in the study (see Table 1). The stakeholders in this study were the HC teams, comprising; older persons (individuals 65 years and older) receiving homecare in ordinary housing, relatives of these older persons (if the person consented), NAs, RNs, OTs, first-line managers, administrative staff, case managers, and authority staff. The stakeholders were invited to co-create knowledge with us regarding how to perceive individual goals in HC. In line with PAAR, the stakeholders became co-researchers.^23^

**Table 1.** An overview of stakeholders.

	Total
Older persons (≤65 years)	29
Relatives	8
Nursing assistants	40
Occupational therapists	8
Registered nurses	11
First line managers	10
Administrative staff	7
Case managers	3
People from authority	2
Total number of stakeholders (*n*):	118

### Data collection

This study is based on 98 datasets, interviews, Focus Group Discussions (FGD),^24^ and Reference groups (see Table 2) gathered by two of the authors (ST, IJ) working with the stakeholders to learn how they reason about individual goals in HC. The data were collected between September 2019 and December 2020 with a pause due to Covid-19 between March and October 2020, which affected the data collection. The project was later canceled due to Covid-19. Guiding questions were formulated in the interviews and FGDs to explore the matter of individual goal-setting in HC: *Describe your goals/the individual’s goals in homecare? Describe the opportunities to work towards these goals in homecare? Are there any obstacles to working towards these goals?* Follow-up questions were used to deepen the knowledge gained. As a first step, interviews were performed within all HC units. In the next step, we returned to the stakeholders and conducted follow-up interviews in three HC units. We also held FGDs within three units to further learn, co-create and deepen our knowledge.^23^ To construct preliminary results, the researchers first compiled data, which were further processed through discussions in reference groups consisting of the HC team. The interviews, FGDs and reference groups were audio recorded and transcribed verbatim.

**Table 2.** An overview of the individual interviews, Focus Group Discussion (FGD) and Reference groups (Ref-gr).

	Individual interviews	FGD 1	FGD 2	FGD 3	FGD 4	FGD 5	FGD 6	FGD 7	FGD 8	Ref-gr 1	Ref-gr 2	Ref-gr 3
Older persons (≤65 years)	29									2	3	1
Relatives	8									2		
Nursing assistants	19	4	2	4		5	6			20	4	4
Occupational therapists	8	1	2	3						2	2	4
Registered nurses	8	2	1	1						3	1	2
First line managers	10	1	1	2				2		1	2	3
Administrative staff	7	1		1					3	2	1	1
Case manager					3							
People from authority				1						3	1	
Total number of stakeholders (*n*):	87	9	6	12	3	5	6	2	3	35	14	15

Due to Covid-19, FGD 4–8 were held through digital video meetings and replaced individual interviews. The stakeholders could participate in one or several interviews, group discussions or references groups.

### Data analyses

**Figure 1.**
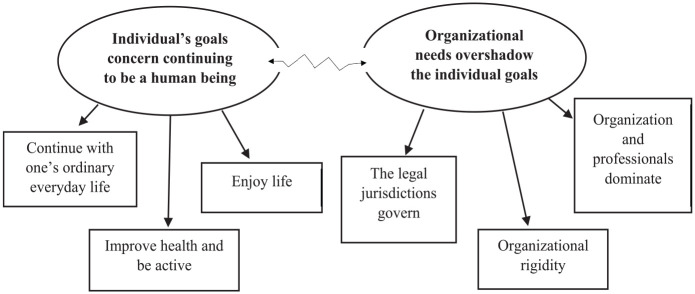
The struggle, opportunities, and obstacles between the individual’s goal and organizational needs within HC.

All data were thematically analyzed^25^ in several steps by two researchers (ST, IJ). First, we listened to the recorded material and read the transcripts several times to become familiar with the material. Second, we coded the material by finding passages of text with similar contents; we divided the transcripts between the researchers, who coded the material separately. Third, similar codes were grouped into themes. This part of the analysis was conducted jointly for greater trustworthiness, to ensure that certain perspectives were not overlooked, and to reduce the risk of bias. Fourth, the themes were compared with the transcripts to ensure that the meaning had not changed during the analytical process. We strived to discern similarities and differences in order to distinguish the themes, and the themes were modified when needed. Fifth, to achieve credibility, all authors refined the themes; different labels were tried and a thematic map was created to illustrate how subthemes were interrelated and gave structure to the main themes (see Figure 1). In the final step, to increase trustworthiness, all the authors tested the themes in relation to their labels and contents, and in relation to the aim of the study.

### Ethical considerations

After considering several ethical matters, we made an ethics application, which was approved by the Regional Ethical Review Board (Dnr: 2019-02575). A conscious choice was made in this project to include persons with complex care needs and cognitive impairments. It can be seen as unethical to include those persons in research, but they are recipients of HC and deserve to be heard. They are able to convey their experiences and knowledge, which makes it unethical to exclude them.^26^ The researchers observed the participants’ body language, expressions, and speech to make sure that they did not display reluctance to participate.

Information about the research was disseminated in several stages. First-line managers disseminated the information at a management meeting. The HC employees received information from researchers at a workplace meeting or another meeting that their managers had booked. Based on previous experience,^23^ we asked the managers to inform potential participants, that is, older persons who would not be harmed by participating (e.g. were not suffering from mental illness or anxiety); information letters were then distributed to those who agreed to participate and written informed consent was obtained from them.

We asked the included older persons whether we could contact their relatives, and after their consent, telephone contact was made. If the relatives agreed, oral and written information about the research was provided and written informed consent was obtained. In sum, all participating stakeholders received oral and written information and provided written informed consent.

## Results

In line with the study design, we reasoned jointly with the stakeholders about the older individuals’ goals in daily life within HC, learning together. All stakeholders talked about all the themes, and described the difference between what the stakeholders defined as the individual’s goals and the organization’s needs within HC as a struggle.

The results are presented according to the two main themes related to the individual’s goals: opportunities for the individual to continue to be a human being; and obstacles to the individual’s goals when organizational needs tend to overshadow the individual’s goals. Six subthemes build on different perspectives within the main themes. Figure 1 illustrates the struggle between these opportunities and obstacles.

### The individual’s goals concerning continuing to be a human being

The first main theme reflects opportunities for the individual to continue to be a human being, where three subthemes give structure to the main theme. *Continue with one’s everyday life* that is, living an ordinary everyday life in various ways and maintaining their individual roles. Furthermore, to continue to be a human being, it was important *to improve health and be more active.* The individuals wanted to improve their physical abilities and they argued that their mental abilities also needed rehabilitation. Another individual goal was *to enjoy life*, which contributes to quality of life.

#### Continue with one’s ordinary everyday life

For the individual to reach his or her goals, the stakeholders emphasized that it was important to understand who the person was earlier in life. They reasoned about the individual’s goal of living an ordinary everyday life in various ways, including getting help with practical chores such as shopping, washing, and cleaning. The older persons may also want to be able to go out for walks or stay at home despite care needs. For example, several older persons had goals such as being able to walk indoors as usual, being as independent as possible so that homecare does not take over their everyday life. The stakeholders described living an ordinary everyday life as having the opportunity to maintain their individual roles, for example, as a grandparent or musician, as well as maintaining their habits by continuing social activities outside the home, as one older person explained:

I’m very active in [an association] and I’ve had an assignment there . . . And now when I got sick . . . There are no opportunities to do that anymore. Now I must try to get started, and possibly go to some meetings as a regular member:

Relatives described the importance of individual goal-setting in homecare to maintain their quality of life as well:

I’ve made changes in my life. But homecare has helped to maintain our quality of life, for sure.

Stakeholders reasoned about the individual goal of having normal conversations, but good conversations can only happen with persons the individual wants to talk with. These conversations allow the individual to maintain relationships and social contacts and to enjoy everyday life.

The stakeholders emphasized conversations with staff as a way for the older individuals to make their voices heard and be able to set goals based on their own wishes, conditions, and interests. The stakeholders believed that older individuals, for example, should be able to choose to have a cup of coffee and converse with the homecare professionals instead of having them do the cleaning, or should be able to schedule home visits around activities such as watching football championships on TV.

#### Improve health and be more active

The stakeholders emphasized the importance of asking about earlier interests or daily routines to help older individuals improve their health and stay active. An NA said:

You must ask “How was your life in the past, what was important to you and what were you interested in?”

Stakeholders said that the individual goal may be to improve physical abilities through exercise, but they pointed out that mental abilities also need rehabilitation. Mental exercise might include learning strategies for remembering things oneself or having intellectual exchanges. An older person gave other examples:

To play cards or have a quiz, it’s about getting a little bit of brain exercise as well. It’s brain gymnastics that are needed . . . Yes, it’s really important.

The stakeholders reasoned about the importance of the physical and social environment where the rehabilitation is carried out, to stimulate the individual’s motivation. Rehabilitation can also be medical, for example, through ensuring wound healing, changing catheters, managing medications, and relieving symptoms. Relief of symptoms might be a goal in itself, and also something that is needed to enable physical and mental rehabilitation.

Stakeholders emphasized the individual goals of becoming healthy and maintaining health by receiving healthcare visits. The older persons wanted to become more mobile and regain previous abilities, with the goals of being able to remove aids and eliminate or reduce dependence on HC staff.

The stakeholders reasoned that the individual’s goals could present an opportunity to enhance motivation—for example, they could be unexpected things, or things that the individual wanted to develop. The goal might be to do something that the older person had not done in a long time or it could also be something the individual was looking forward to, for example having a sex partner, getting married, or learning a language.

#### To enjoy life

Stakeholders thought that as the individuals’ need for help increased due to declining health, their goal might be to put their energy into what gives them joy and contributes to quality of life. To save energy for the activities that bring meaning to life, the individual might need help with some daily activities, even though the individual has the physical ability to perform them. A first-line manager described the situation as follows:

But it can also be like when you get older, then you don’t have as much energy as you had when you were younger and then . . . I want to put my energy into showering myself. But then someone has to help me to shop because once I’ve showered, I’m completely exhausted. For me, the goal is what gives a better quality of life.

All stakeholders emphasized the existential perspectives and described the importance of meeting spiritual needs by showing the older persons that you care, in the moment. The stakeholders believed that the individual’s goal shows what it is to be a human being. In line with that, they emphasized that one goal is not enough. The individual needs to have multiple goals to manage daily life. The goals were described as everyday activities, such as making phone calls, putting on stockings, going to a funeral, cleaning, or walking the dog, as well as having opportunities to enhance the individual’s mental health. These are activities that allow the individual to be active and to experience a high quality of life. By doing activities based on their interests, life becomes valuable and meaningful for the older persons. Stakeholders emphasized that the standards of living differ among all individuals, arguing for the ability to have goals that allow for a “golden” element, brightening up everyday life through activities that make the older individual feel good. They described such activities as having manicures, holding hands, picking mushrooms, listening to music, buying ice cream, or having an evening sandwich. The stakeholders emphasized the possibility of deep conversations occurring during these activities.

As the loneliness of older persons is great, a goal for the older person should be to counteract loneliness. The stakeholders emphasized time for conversations as an opportunity to allow the older individuals to feel important; they said that the conversations should be conducted out of a sense of compassion, not of duty. One older person described the situation thus:

And then there are individuals who after everything they’ve done, they sit down and talk a little bit. They notice when you are sad or concerned. They sit down and talk. This is a great way to help an older person alleviate loneliness, because loneliness is something terrible . . . They sit and listen to you and show . . . not a sense of duty, but love.

### Organizational needs overshadow the individual’s goals

The second main theme reflects the struggle occurring when organizational needs overshadow the individual’s goals, where three subthemes give structure to the main theme. M*ultiple legal jurisdictions govern*, and the individual’s goals “falls between the cracks” and are forgotten. *Organizational rigidity* fails to accommodate individual needs and wishes; rather, the organization’s finances and resources create the framework. Additionally, *the organization and the professionals dominate* and the individual goals are overshadowed by professional goals.

#### The legal jurisdictions govern

Stakeholders reasoned about obstacles to realizing the individual’s goals, and it appears that individuals risk “falling between the chairs” when several different legal jurisdictions must work together. Professionals say that while the case manager may perceive a certain goal, the individual goal is said to be something else by the NAs, who observe different needs and goals when they get to know the individuals. One RN commented:

Then I think a big obstacle is that we do more than what’s written in the assignment. And then you become the culprit. For there has to be a decision about everything, instead of actually solving that stuff and getting quality.

The stakeholders described how obtaining HC is a time-consuming bureaucratic process. The older individuals said that their goals might be to get more HC time, but when the case manager does not grant time based on the Social Services Act, OTs and RNs circumvent the system and grant extra time through the Health and Medical Services Act. The professionals argued that this is the wrong way to use the system. When the time granted does not match the individual goals, distrust of the organization arises. The professionals also emphasized that another obstacle is that the assignments are not detailed enough, lacking descriptions of what the individuals can do by themselves to achieve their goals.

The stakeholders argued that crucial decisions such as following up individual goals are delayed, resulting in the risk of missed rehabilitation and, consequently, deteriorated health. The stakeholders described how older persons often got a lot of support immediately after a hospital stay, intended to restore their function and, ultimately, reduce the interventions provided. However, when follow-up of this extra support is delayed, there is the risk of individuals getting used to the extra help and losing functions and motivation.

#### The organization is rigid

The stakeholders reasoned that it is an obstacle when the organization decides which staff should make the home visits regardless of the older individual’s wishes. For example, stakeholders said that when the individuals do not feel confident with the staff, they may have difficulties challenging themselves in exercise or feel uncomfortable when they shower, leading them to give up on their goals and refrain from interventions. The stakeholders also described how an individual may have different goals depending on what profession makes the home visit.

The organization is structured in silos, with everyone focusing on their own jobs, not on the older persons’ individual goals. This leads to difficulties cooperating with other professions from other parts of the organization or from outside municipal HC, as an OT described:

Team collaboration is number one . . . We have a fairly small unit, and the cooperation between the nurse and the homecare service works quite well. The case manager of the case is the more difficult one to cooperate with. Physiotherapists and primary care are difficult to work with. Even with the hospital . . . More team collaboration—I think that must improve.

#### The organizational and the professionals dominate

The older persons questioned whether they really needed to have a goal, and questioned whether the goal is actually for the organization’s interests. Stakeholders described how the stipulated chores can be the same as the individual goals, meaning that, from an organizational perspective, the goals concern what household chores and interventions can be offered. Stakeholders reasoned that the case manager’s description of the goals of the assignment can be broad and difficult to understand, impeding the achievement of the individual’s goals. One obstacle is that the NAs do not have sufficient time to sit down with the older individuals and analyze their goals, and to clarify their goals for everyone in the team. The stakeholders stressed that one reason is an economic compensation model that does not allow sufficient time for the NAs to create a relationship and talk about the goals. Furthermore, when the individual’s goals are not clear to everybody, it is hard for the professionals to cooperate on an equal footing. This implies that it is the organization’s finances and resources that constitute the framework, negatively affecting the individual’s goals. As a first-line manager reflected:

I think we’re pretty frustrated because we don’t have balanced finances, we don’t have the conditions . . . this first conversation with a customer . . . it’s certainly an hour and the time for documentation is not included—we don’t have the money for it.

Stakeholders said that it becomes an obstacle when the organization has goals and guidelines that promise a lot. It may not be the individual’s goals, but rather what the organization can offer so that the individual can stay at home that is described as the older person’s goals. Professionals also told how the individual’s goals are based on the profession’s assigned tasks, not on the older person’s wishes. The organization’s goal is used to convey information about the individual’s health to other professions, so the focus is on the intervention rather than the individual goals, for example, ensuring medication administration or wound healing. Older persons are very familiar with the conditions of HC and try to facilitate the NAs’ daily work rather than expressing their own goals and wishes, and the stakeholders identified this as an obstacle to individual goal-setting. One older person described the situation:

So I think you probably need to give the staff a more tolerable life, through better conditions in terms of pay and training. I need help from the homecare service, but you get involved in their schedule and [with] hourly employees who are working despite [being on] vacation. There will be people who don’t really want to be here [who are] at work.

The stakeholders noted that whereas the organization is task oriented with a focus on time and finances, mental health and pleasure considerations are missing from the assignments. The professionals described the organization’s and older individuals’ different definitions of quality of life as constituting an obstacle, resulting in the deprioritization of a content-rich, joyful life. This discrepancy can also lead to the individual’s expectations of HC not being in line with the organization’s goals.

## Discussion

The results indicate a struggle between the goals of the individual, who is striving to continue being human, and the organization’s needs, a struggle in which the latter overshadow the former. The discussion is centering on the stakeholders reasoning about individual goalsetting, and is divided into the two main themes and six subthemes presented above.

### Individual’s goals concerning continuing to be a human being

We learned from stakeholders that the older individual’s goals are to *continuing with one’s ordinary everyday life* and maintaining their role of, for example, a grandparent or musician. The stakeholders taught us about the importance of getting to understand who the person was to be able to give her or him individual support. They stated that the individual needs to be allowed to determine depending on how she or he feels on a given day—perhaps the individual may wish to drink coffee and talk with the NA instead of having them do the cleaning. Such freedom to exert control is important so that the individual can feel like a human being. This is in line with flow theory, according to which a sense of control and security, for example, to shape one’s habits and routines and express different roles, can strengthen the subject’s self-confidence and self-esteem.^27^ This is also consistent with Wilcock and Hocking,^28^ who stated that the person must undertake activities in order to feel good. Expressing oneself through one’s roles is a way to be human, and different roles might motivate persons to undertake different activities. We also learned from stakeholders that the older individual’s goals are to *improve health and be more active*. To maintain an ordinary life is important not only for the individuals to stay in their own homes, aging in place^4–6^ but also in line with public health priorities.^1,7^ Furthermore, it is important to improve the individual’s health in medical terms by, for example, relieving symptoms and rehabilitating physical and mental health. Additionally, we also learned that another way to strengthen public health is to support the individual’s goals concerning *enjoying life* and being human, despite impaired health. The stakeholders emphasized that time for conversations was an opportunity to allow the older individuals to feel important; they described that the conversations should be conducted out of a sense of compassion. The stakeholders taught us that the existential perspective of the individual’s goals were important in order to meet emotional and spiritual needs. HC professionals may need to sit down for a moment, converse with the older individuals, listening to them and show that they care. This would alleviate their loneliness and, at best, provide a “golden” moment brightening up their everyday. This is also in line with flow theory, according to which one must focus on the positive and healthy to deal with reactions to difficulties and to experience happiness.^27^ When a person experiences an activity as meaningful and within her/his capacities, a feeling of flow arises and the person feels good.^27^ Therefore, the older individual’s goals, when receiving HC, should incorporate social goals and activities that not only address bodily needs, but also are things that the individual finds enjoyable. This is consistent with the dimensions of the WHO’s^3^ definition of health as when a person has physical, mental, and social wellbeing. It is also in line with the WHO’s stipulation that healthy aging and the overall goal of HC entails maintaining individual health. The older individual’s HC goal must achieve a balance between rest, activities, and exercise in order to improve physical and mental abilities. The goals must also be able to change over time and be adapted to the individual’s context and wishes, so that the older person can feel fully human. It is important to empower older persons to do things that they are capable of,^29^ to change the approach of HC staff from “doing for” to “doing with” so that the individuals can regain previous routines and habits, a process that the individuals and staff undergo together.^30^ To ensure public health, it is important that aging with a need for help not be seen as a failure.^31^

### Organizational needs overshadow the individual goals

We also learned that there are several obstacles impeding individuals from pursuing their goals. The organization in HC controls and overshadows the individuals and their goals, and HC tends to be rigid and task oriented. The individuals’ goals are governed by *the legal jurisdiction*, and it is others, such as professionals, who make decisions for individual older persons. In Sweden, this could be because municipal HC is politically controlled and organized and is affected by how society values the older people. McDonald et al.^32^ showed that it is easy to forget that older persons want to live life to the fullest, through being involved in society and performing everyday routines at home, and that aging can be misconstrued as being powerless. Here, we have learned that excessive attention to older person’s problems could obstruct identification of what might bring joy to them in everyday life. Worldwide, society is affected by age-based discrimination,^29^ which leads to poorer public health, social isolation, premature death, and considerable financial costs.^33^ However, there are struggles in which those who need help do not get it according to their individual needs because of the organizational requirement to save money. The stakeholders described a lack of HC time, with HC focused on what tasks homecare could offer, not the individual’s goals. This situation recalls the description of a stupid organization in which narrow, uniform thinking with a focus on finance and management means that no one dares to question the organization.^34^

Stakeholders emphasized that the focus on time and finances became an obstacle to reach individual’s goals related to mental health and intellectual stimulation. Further, the organization was described as an obstacle to work supporting older persons’ dignity and well-being^35^; it has been claimed that HC has insufficient organizational resources to ensure safe care.^36^ The human aspect has been lost as new public management reforms have affected Swedish HC, creating a focus on time and the organizational goal of cost efficiency.^5^ Healthcare seems to value keeping the individual alive as long as possible but has forgotten to emphasize quality of life as well.^32^

The stakeholders reasoned that *organizational rigidity* it is an obstacle when the organization decides which staff should make the home visits regardless of the older individual’s wishes. The individuals do not feel confident with the staff, leading them to give up on their goals and refrain from interventions. Today, older individuals cannot exert control over their HC; however, since they pay a fee for the service, there should be greater focus on its content and quality.^37^ The goals of HC need to be meaningful and challenging, and set by the older person, who will thus “own” them. It may be impossible to achieve any HC goal that the targeted individual, as a human, does not “own” or control.^27^ Today, there is a struggle between the older individual, who is not given the opportunity to be fully human, and the organization, whose priorities overshadow the individual’s everyday life. Given the lack of resources to promote older persons’ mental health, it has become normal for them to feel lonely, anxious, and depressed. Those who assess individual needs need to challenge structures such as ageism and ensure that older persons have the same rights as do other citizens.^29,37–39^ This is of great significance for public health.

We also learned that the *organization and the professionals dominate over the individual’s goals.* The older individuals told us that an obstacle is when they cannot express their own goals, there is a risk that the professionals may set the goals. It may also be the case that different professions have different goals and interventions. In this study, we learned that individuals insufficiently participated in the process of goal-setting. Jokstad et al.^30^ described how professionals sometimes abuse their power when they want to start HC as fast as possible, setting goals without the individual’s participation. Another obstacle might be that joint goal-setting with the older individual demands time and some advance knowledge of the person. Regardless of the professions in the team, older individuals’ involvement and collaboration within the team must increase to make them feel secure.^30,40^ HC is supposed to enable person-centered reablement, where the individual’s goals are clear and support in the home environment allows the individual to regain their everyday life.^41^ It requires that healthcare professionals have goal- and person-oriented communication skills, and encourage the older person’s participation in mapping their resources and needs, which leads to the formulation of goals.^42^ There should also be procedures in place to allow individuals to give and receive explicit feedback on their goals.^27^ The struggle must stop. We learned that the most important way to increase the individual’s participation in goal-setting in HC is to ask the older person what is most important to her/him. This would acknowledge their full humanity and prevent the organization from overshadowing and controlling the individuals and their goals.

### The study’s strengths and limitations

#### Strengths

A strength is that we collaborated with the stakeholders as co-researchers and used several data collection methods, namely, interviews, FGDs, and reference groups. We reviewed what stakeholders had said earlier and, on that basis, repeated interviews. We also held FGDs, which increased the trustworthiness of the study. The iterative process whereby we collaboratively built practical knowledge together with the stakeholders contributed to the trustworthiness of the results.^21^ Another strength is that data were collected in various contexts, ranging from central urban areas to the countryside and we met with participants of several different professions.

#### Limitations

A limitation is that we did not meet with individuals from socioeconomically vulnerable areas, where individual goals might differ according to other cultural and ethical perspectives. Another limitation is that we could have gone back to the stakeholders to a greater extent to learn more. The data collection also varied in the different units due to Covid-19 and we were forced to take a break due to Covid-19.

## Conclusion

In conclusion, we learned that the older person receiving HC must have the same rights to be human, with their own goals, as other citizens, which is in line with public health goals. The organization needs to be flexible and reduce the focus on time and economic considerations, in favor of the older individual’s goals and, thereby, quality of life. The older individual should be facilitated to be fully human, possessing control, confidence, and self-esteem; balance in daily life supported by challenging meaningful goals.

## Recommendation

The organization must have a power shift of focus, from economics, to the older individual’s goals. Through person-centered reablement, the team members must have close collaboration striving for the same goals, that is, the older individual’s goals. The professionals must have person-oriented communication skills, the ability to listen, and to perceive the individual’s goals. The individuals themselves must set and evaluate their goals. There is a need to focus on the older person′s goals, to succeed with that the professional must ask the individual; what is important to you?
